# Beyond the Lab: Empirically Supported Treatments in the Real World

**DOI:** 10.3389/fpsyg.2020.01969

**Published:** 2020-08-11

**Authors:** Renee A. Schneider, Joseph R. Grasso, Shih Yin Chen, Connie Chen, Erin D. Reilly, Bob Kocher

**Affiliations:** ^1^Lyra Health, Burlingame, CA, United States; ^2^Department of General Internal Medicine, University of California, San Francisco, San Francisco, CA, United States; ^3^Department of Psychiatry, University of Massachusetts System, Boston, MA, United States; ^4^School of Medicine, Stanford University, Stanford, CA, United States

**Keywords:** empirically supported treatment, evidence-based treatment, depression, anxiety, mental health problems

## Abstract

Laboratory studies of empirically supported treatments (ESTs) for mental health problems achieve much higher rates of clinical improvement than has been observed following treatment in the community. This discrepancy is likely to due to limited reliance on ESTs by therapists outside of academia. Concerns about the generalizability of ESTs to patients in the community, who may have comorbid problems, likely limit rates of adoption. The present study examined the impact of ESTs delivered in the real-world for 1,256 adults who received services through an employee assistance program specializing in the delivery of ESTs. Rates of anxiety and depression decreased significantly, following treatment with an EST, and 898 (71.5%) patients demonstrated reliable improvement. Even among patients comorbid for depression and anxiety at baseline, over half reported reliable improvement in both disorders. Findings suggest ESTs can be effectively delivered outside of academic RCTs. However, additional research is needed to understand and overcome barriers to disseminating ESTs to the broader community.

Empirically supported therapies (ESTs) are behavioral health interventions that have been rigorously tested in randomized controlled trials (RCTs) or a series of well-designed single-subject experiments and have demonstrated efficacy when compared to a control or active treatment condition (Chambless and Hollon, 1998; Tolin et al., 2015). ESTs have been developed for a range of behavioral health problems, including the most common disorders, depression and anxiety. In particular, cognitive behavioral therapy (CBT), cognitive therapy (CT), and interpersonal psychotherapy have all demonstrated efficacy in the treatment of moderate or severe depression, with evidence suggesting that CBT and CT may be as efficacious as antidepressant medication (Shapiro et al., 1994; Gloaguen et al., 1998; DeRubeis et al., 2005). Multiple meta-analyses also support the efficacy of CBT or behavioral therapies for the treatment of anxiety disorders, including panic, obsessive-compulsive disorder, social anxiety, post-traumatic stress disorder, and generalized anxiety disorder (Deacon and Abramowitz, 2004).

Reliable improvement, defined as symptom improvement not accounted for by measurement error alone (Jacobson and Truax, 1991), is the standard by which ESTs are often measured. Laboratory studies of ESTs, in which efficacy is tested in an RCT, demonstrate rates of reliable improvement at or greater than 50% (Hofmann et al., 2012; Loerinc et al., 2015). However, findings indicate that among adults treated with psychotherapy under naturalistic conditions in the community, fewer than 30% achieve reliable improvement (Hansen et al., 2002).

One explanation for the discrepancy in observed outcomes may be due to limited adoption of ESTs by providers in the community. Despite efforts to disseminate ESTs more broadly, they are underutilized outside of academia (Stewart and Chambless, 2007). Concerns about the external validity of RCTs are frequently cited by clinicians in the community (Nelson and Steele, 2007; Kazdin, 2008), who worry that ESTs are infeasible to implement in real-world settings (Addis et al., 1999) and that subjects in RCTs differ in important ways from patients in community settings (Westen and Morrison, 2001). In particular, it has been hypothesized that including patients with psychological comorbidities, which tends to be the norm in real-world clinical settings, could reduce the impact of ESTs (Shadish et al., 2000).

Unfortunately, treatment studies often focus only on a single diagnosis, with comorbid psychiatric diagnoses considered exclusion criteria, thus diminishing their application to real-world settings (Goldstein-Piekarski et al., 2016). There are only a small number of RCTs examining EST effectiveness with more real-world–like samples. For example, Craigie and Nathan (2009) found that both individual and group CBT could be effectively used to treat depression in a sample of adult community outpatients with high rates of comorbidity. Barlow et al. (2017) similarly demonstrated that ESTs could be effective in the treatment of adult anxiety disorders when applied to patients with multiple psychiatric diagnoses.

However, other studies on the generalizability of ESTs have produced mixed results, with some research suggesting that ESTs are less potent when delivered outside of more controlled settings (e.g., Weisz et al., 2006; Jonsson et al., 2015). Consistent with these findings, it has been suggested that as study conditions more closely resemble the real world, the efficacy of ESTs may diminish (Westen and Morrison, 2001; Stewart and Chambless, 2009). For example, when providers are not required to use a treatment manual or when treatment fidelity is not measured, CBT may be less potent, as therapists may “drift” from standardized practices (Waller, 2009). Further, therapists in well-controlled RCTs often receive more intensive training in ESTs and clinical consultation than therapists practicing in the community (Weisz et al., 2006; Becker and Stirman, 2011), which may affect treatment fidelity and outcomes.

The EST literature has been criticized for focusing on efficacy in tightly controlled clinical trials at the expense of real-world generalizability (Tolin et al., 2015), and there are few studies that examine the efficacy of ESTs under more naturalistic conditions. In particular, only a handful of studies have looked at how adult patients with psychological comorbidities fare when treated with an EST. In addition, most studies of ESTs rely on a randomized controlled design, which may limit the generalizability of findings as not all patients are willing to be randomized to conditions (Tolin et al., 2015). Studies of the portability of ESTs outside of academia are often conducted in the context of extensive clinical training and oversight, something that is generally lacking in the community. In revising the criteria for ESTs, Tolin et al. (2015) encouraged research that (1) did not involve randomizing subjects to conditions, (2) was conducted by clinicians outside of academia, and (3) involved patients with behavioral health comorbidities.

This present study applies a retrospective design to build on the criteria outlined by Tolin et al. (2015) to better understand whether ESTs for behavioral health problems are effective under real-world conditions. We examined rates of reliable improvement among patients with depression or anxiety who received an EST from a community therapist. Because research on the efficacy of ESTs for patients with psychological comorbidities is limited, we also report separately rates of reliable improvement among patients who started treatment with both depression and anxiety.

## Methods

### Participants

**FIGURE 1:**
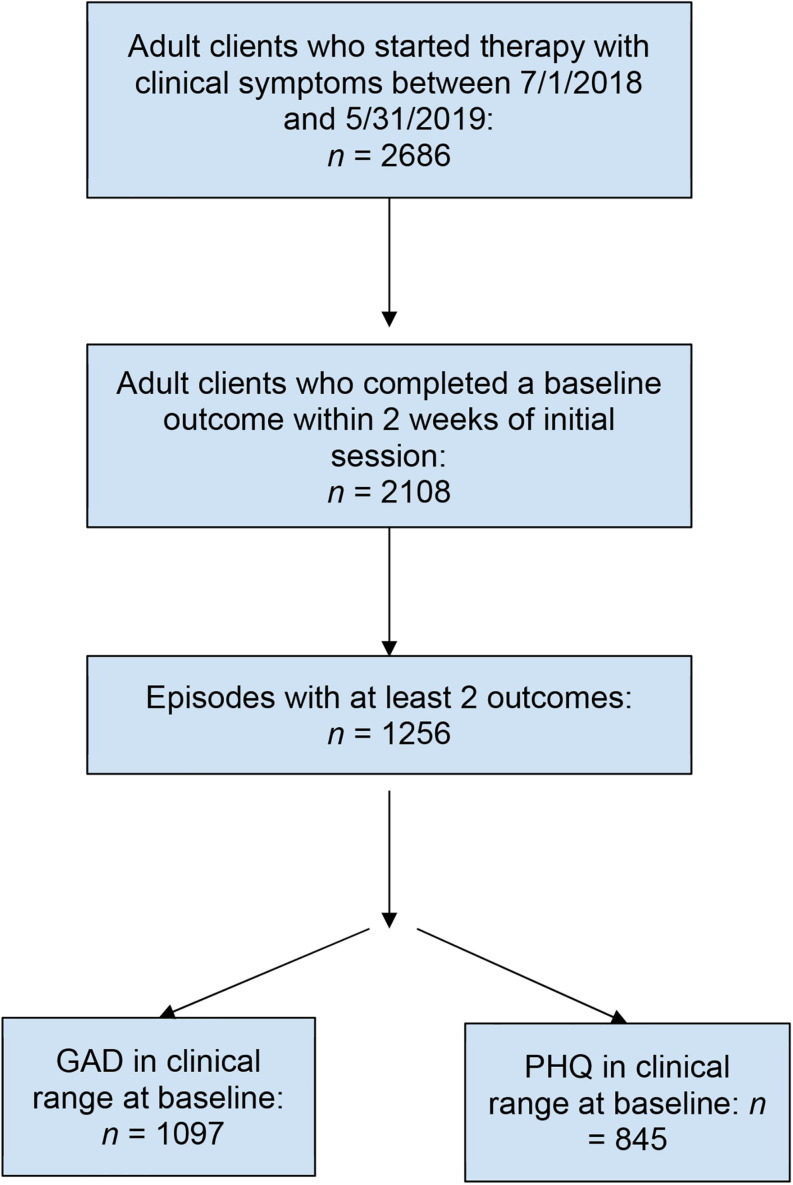
Participant flow through the study.

Adult patients, 18 years or older, who started individual therapy between July 1, 2018 and May 31, 2019, were included in the present study. All patients in the study were referred to a community therapist by an employee assistance program (EAP) that partners with therapists who utilize ESTs. Patients were employees or dependents of customers who had purchased the EAP. Patients were included in the study if they scored in the clinical range on a measure of depression or anxiety and completed a baseline assessment within 2 weeks of their first appointment (Figure 1). Patients were sent electronically secure assessment questionnaires every 4 weeks following their first appointment. Baseline assessments were compared to the most recent assessment to estimate the impact of treatment. This study was deemed exempt from human patients review by the Western Institutional Review Board.

### Therapists

All therapists in the present study were in community private or group practices and had agreed to join the network of an EAP that specializes in referring patients to providers who practice ESTs. Prior to joining the EAP network, a vetting team reviewed each provider’s public presence (e.g., website) and application, if available, to determine whether they likely utilized ESTs. Those providers who passed this initial step were invited to participate in a clinical vetting interview designed to test their knowledge of and ability to apply ESTs. Sample components of the clinical interview include asking prospective therapists about their theoretical orientation, the therapies and interventions they use, how they measure treatment progress, the average length of treatment, and how they adapt treatment plans based on a patient’s response to treatment. In particular, we were looking for providers who used ESTs as defined by Chambless and Hollon (1998) and Tolin et al. (2015), used validated measures to assess treatment progress and outcomes, and practiced short-term therapy in contrast to treatments of indeterminate length. Only those therapists who passed the rigorous clinical vetting interview were invited to join the network. Historically, approximately 5% of providers who applied to join the network have passed the review and vetting interview. Providers included in the study were compensated monetarily, as per standard community practice.

### Measures

Assessments consisted of the Patient Health Questionnaire 9 (PHQ-9) and Generalized Anxiety Disorder scale (GAD-7), well-validated measures of depression and anxiety (Kroenke et al., 2001; Spitzer et al., 2006; Plummer et al., 2016). The PHQ-9 includes nine items rated on a 4-point scale, with scores ranging from 0 to a maximum of 27. The GAD-7 includes seven items rated on a 4-point scale, with scores ranging from 0 to 21. Both measures have been shown to be sensitive to treatment changes over time in psychiatric populations (Beard and Björgvinsson, 2014). For inclusion in the study, a clinical cutoff of 10 was used for the PHQ-9, as research suggests that patients who score at or greater than 10 are very likely to meet the criteria for major depression (Kroenke et al., 2001). A clinical cutoff of 8 was used for the GAD-7, as research suggests that scores at or greater than 8 are highly likely to correspond to an anxiety disorder diagnosis (Kroenke et al., 2007; Plummer et al., 2016).

### Analyses

Multiple regression analyses were conducted to examine a possible association between number of sessions and reduction in PHQ-9 and GAD-7 scores, respectively, after controlling for baseline scores. Paired *t-*tests were used to test differences in PHQ-9 and GAD-7 scores between baseline and follow-up. For each measure, we compared the baseline assessment score to the last available assessment and calculated Cohen’s *d*_*rm*_, a conservative measure of effect size for within-subjects designs that controls for correlation between measurements (Lakens, 2013). We calculated the number of patients who demonstrated reliable improvement on either measure, using the RC index proposed by Jacobson and Truax (1991). The RC index allowed us to determine whether a patient made substantial improvement in symptomatology, beyond what could be attributed to measurement error. Consistent with previous research (Gyani et al., 2013), we used an RC index of 6 for the PHQ-9 and 4 for the GAD-7. We also calculated the percentage of patients who recovered, meaning they moved from the clinical range to below the clinical cutoff on either measure. In addition, we calculated the number of patients who demonstrated reliable recovery (Gyani et al., 2013) in that they both demonstrated reliable improvement and recovered on either the PHQ-9 or GAD-7. Finally, to better assess the impact of treatment on patients with psychological comorbidity, rates of reliable improvement and recovery are reported separately for patients who started in the clinical range on both the PHQ-9 and GAD-7.

## Results

Of the 1,256 patients included in the analyses, 54.3% (*n* = 682) identified as female, 33.3% (*n* = 418) identified as male, 12.3% (*n* = 155) did not specify gender, and gender was missing for 1 patient. Sixty-eight percent of patients were between the ages of 18 and 34 years. The mean age of patients was 32.8 (*SD* = 8.7) years (Table 1).

**TABLE 1:** Characteristics of subjects and therapists.

Subjects *n* (%)		
Gender	Female	682 (54.3)
	Male	418 (33.3)
	Unspecified	155 (12.3)
	Missing	1 (0.1)
Age group, years	18–24	153 (12.2)
	24–34	699 (55.7)
	35–44	275 (21.9)
	45–54	86 (6.8)
	55–64	41 (3.3)
	=65	2 (0.2)
Clinical presentation	PHQ-9 =10	845 (67.3)
	GAD-7 = 8	1,097(87.3)
	PHQ-9 and GAD-7 in clinical range	686 (54.6)
**Therapists *n* (%)**		
Therapist credential	Doctoral level	242 (43.3)
	Master’s level	317 (56.7)
Therapist years of experience	<5	137 (24.5)
	5–10	195 (34.9)
	10–15	108 (19.3)
	15–20	64 (11.4)
	>20	52 (9.3)
	Missing	3 (0.5)

The 1,256 patients saw 559 separate therapists (Table 1). On average, each therapist saw 2.1 (*SD* = 1.6) patients. Approximately 43.3% of therapists had a doctoral degree; 56.7% of therapists had a master’s degree (e.g., LMFT, LCSW, LPCC). Therapists delivered ESTs, as per their normal practice, with the exception that patients could receive up to a pre-specified number of session visits per calendar year, with the maximum number of sessions being 25. The average number of sessions delivered across the course of treatment was 9.4 (*SD* = 7.13). The average number of weeks patients spent in treatment was 13.1 (*SD* = 10.4). The number of sessions was inversely correlated with depression and anxiety at follow-up, in that patients with more sessions showed greater improvement on the PHQ-9 [β = −0.05, *t*(1253) = 2.60, *p* = 0.01] and the GAD-7 [β = −0.05, *t*(1094) = 2.65, *p* < 0.01], after controlling for baseline scores.

Independent-samples *t*-tests were conducted to compare baseline severities on the GAD-7 and PHQ-9 for patients who did and did not complete a second outcome assessment. In terms of baseline PHQ-9 scores, there was no significant difference in severity for patients who completed a second outcome (mean = 14.30, *SD* = 3.79) and patients who did not complete a second outcome (mean = 14.65, *SD* = 3.83); *t*(1418) = −1.69, *p* = 0.09. Similarly, there was not a significant difference in the severity of baseline GAD-7 scores for patients who completed a second outcome (mean = 12.69, *SD* = 3.61) and patients who did not complete a second outcome (mean = 12.70, *SD* = 3.62); *t*(1860) = −0.05, *p* = 0.96.

### Depression Symptoms

Of the 1,256 patients, 845 (67.3%) scored in the clinical range on the PHQ-9 at baseline. The baseline average score on the PHQ-9 was 14.30 (*SD* = 3.79), corresponding to the moderate range of depression severity. Results of paired *t*-tests revealed that among patients who started in the clinical range, depression scores decreased significantly between baseline and follow-up (mean = 7.58, *SD* = 4.45), with patients improving an average of 6.7 points on the PHQ-9 [95% confidence interval (CI), 6.4–7.1], *t*(844) = 39.00, *p* < 0.001. Cohen’s *d* suggested a large treatment effect size on depression (*d*_*rm*_ = 1.62). Reliable improvement in depression scores was observed in 509 patients (60.2%), and 602 patients (71.2%) recovered on the PHQ-9 (Table 2). A total of 448 patients (53.0%) demonstrated reliable recovery on the PHQ-9, defined as meeting criteria for both reliable improvement and recovery.

**TABLE 2:** Clinical change and recovery.

*n* (%)	Baseline positive screening for depression (*n* = 845)	Baseline positive screening for anxiety (*n* = 1,097)	Baseline positive screening for depression or anxiety (*n* = 1,256)	Baseline positive screening for depression and anxiety (*n* = 686)
Reliable improvement	509 (60.2)	750 (68.4)	898 (71.5)	361 (52.6)
Recovery	602 (71.2)	682 (62.2)	934 (74.4)	350 (51.0)
Reliable recovery*	448 (53.0)	595 (54.2)	774 (61.6)	269 (39.2)
Reliable improvement or recovery	663 (78.5)	837 (76.3)	1,039(82.7)	442 (64.4)

* *Reliable recovery is defined as reliable improvement and recovery.*

### Anxiety Symptoms

At baseline, 1,097 patients (87.3%) scored in the clinical range on the GAD-7. The average baseline score on the GAD-7 was 12.69 (*SD* = 3.61), corresponding to the moderate range of anxiety severity. Anxiety scores decreased an average of 5.6 points (95% CI, 5.3–5.9) between baseline and follow-up (mean = 7.07, *SD* = 4.35), and results of paired *t*-tests revealed that this difference was statistically significant, *t*(1096) = 38.66, *p* < 0.001. Cohen’s *d* suggested a large treatment effect size on anxiety (*d*_*rm*_ = 1.40). Of the 1,097 patients with clinical scores on the GAD-7 at baseline, 750 (68.4%) met the criteria for reliable improvement, and 682 patients (62.2%) recovered (Table 2). Reliable recovery from anxiety was observed in 595 patients (54.2%).

### Depression and Anxiety Symptoms

A total of 686 patients (54.6%) scored in the clinical range on both measures at baseline, suggesting they were comorbid for depression and anxiety. Of these, 361 patients (52.6%) showed reliable improvement on both measures, and 350 (51.0%) recovered on both measures (Table 2). Approximately 269 (39.2%) of the 686 patients demonstrated reliable recovery from both depression and anxiety.

## Discussion

Findings presented here demonstrate that ESTs can be efficacious under real-world conditions and deliver results that are comparable to those observed in RCTs (e.g., Hofmann et al., 2012). Among patients receiving an EST from a community provider, levels of depression and anxiety significantly decreased over the course of treatment. Of note, more than half of patients who were comorbid for depression and anxiety at baseline made meaningful improvement in both areas. In utilizing the criteria outlined by Tolin et al. (2015), this study further supports the efficacy of ESTs and extends their usefulness to settings outside of academia.

It is widely known that a gap exists between academia and real-world clinical practice, with the majority of providers in the community relying on prior experience and professional preferences, rather than research data, to inform their clinical decisions (Stewart and Chambless, 2007; Lilienfeld et al., 2013). One reason that community providers cite for rejecting ESTs is a concern that subjects in RCTs are not representative of patients in the real world who may have higher rates of comorbidity (Shafran et al., 2009). Contrary to this hypothesis, findings presented here are consistent with previous research (Craigie and Nathan, 2009; Barlow et al., 2017) and suggest that, even for patients with psychiatric comorbidities, ESTs can produce significant symptom reduction. Further, treatment effectiveness was comparable to efficacy rates seen in RCTs (e.g., Hofmann et al., 2012) despite the lack of clinical oversight and standardized training in ESTs that are characteristic of most studies examining EST effectiveness in the community.

Some limitations to the present study should be considered. In particular, there was no measure of treatment fidelity, so we cannot be certain whether ESTs were delivered with strict adherence to treatment manuals. However, this limitation allowed us to examine how ESTs perform when delivered under more naturalistic conditions by therapists with heterogeneous training in ESTs and use of varied ESTs. It is also possible that therapists combined elements of different ESTs, and in fact, the research suggests that this approach may be associated with better outcomes for patients with psychological comorbidities (Chorpita et al., 2013).

It would have been helpful to capture what other comorbid behavioral health conditions patients may have been experiencing and more specific details on the types of anxiety or depressive disorders patients had. In addition, we did not account for any other treatments patients may have been receiving that could also have produced a change in depression or anxiety. Further, because this was a naturalistic study, the timing of when baseline and follow-up measures were completed varied across patients. It is possible that the last outcome measure we have for some patients was collected midtreatment, and we would have seen even higher rates of reliable improvement and recovery if all patients completed the final outcome survey immediately after the end of treatment. It should also be noted that this was not an intent-to-treat analysis, and there may have been important differences between those patients who completed outcomes assessments and those who did not or those who dropped out of treatment prematurely, possibly skewing findings in a more positive direction.

Finally, because of a possible therapist selection bias, it is unknown whether these results are generalizable to ESTs delivered by community providers who have not undergone extensive vetting. In the majority of studies testing the generalizability of ESTs beyond academia or with patients who have psychological comorbidities, providers receive considerable clinical training, and treatment fidelity is measured in an ongoing way (e.g., Weisz et al., 2006; Barlow et al., 2017). Additional research is therefore needed to determine whether most providers can deliver ESTs in the community without substantial clinical vetting or oversight.

Further research is also needed to understand which ESTs are most easily transported to community settings and what modifications, if any, therapists in the community must make to improve patient acceptability. In traditional research studies, there may be a self-selection bias favoring patients who are interested in more structured or novel interventions and who are better-educated around what ESTs typically entail. Patients in the community may be less familiar with therapy, in general, and likely have very different expectations for therapy relative to those participating in a research study at an academic center. Therapists in the community may be adapting ESTs to make them more appealing to patients, and an understanding of these adaptations could improve efforts to disseminate ESTs more broadly.

Despite the limitations, this study addresses an important gap in the empirical research on the external validity of ESTs. Given that research demonstrates that ESTs are effective outside of academia (e.g., Weisz et al., 2006; Craigie and Nathan, 2009), translational studies aimed at understanding and overcoming barriers to the adoption of ESTs in the real world are an important next step. Understanding what changes therapists in the community may make to ESTs to improve acceptability and how those changes affect treatment efficacy may enhance dissemination of ESTs outside of academic settings and increase the sustainability of EST implementation in clinical practice.

## Data Availability Statement

All datasets generated for this study are included in the article/supplementary material, further inquiries can be directed to the corresponding author.

## Ethics Statement

The study was granted exempt status by the Western IRB. Written informed consent from the participants was not required to participate in this study in accordance with the national legislation and the institutional requirements.

## Author Contributions

RS wrote the initial draft of the manuscript. JG, CC, ER, and BK contributed to the development and editing of the manuscript. SC did the statistical analyses. All authors contributed to the article and approved the submitted version.

## Conflict of Interest

RS, JG, SC, BK, and CC were employed by or have equity in Lyra Health, Inc. The authors declare that this study received funding from Lyra Health, Inc. The funder had the following involvement in the study: funding of data collection process; employment of co-authors who designed the study, conducted analyses, and prepared the manuscript.

## References

[B1] Addis M. E. Wade W. A. Hatgis C. (1999). Barriers to dissemination of evidence-based practices: addressing practitioners’ concerns about manual-based psychotherapies. *Clin. Psychol.* 4 430–441. 10.1093/clipsy.6.4.430

[B2] Barlow D. H. Farchione T. J. Bullis J. R. Gallagher M. W. Murray-Latin H. Sauer-Zavala S. (2017). The unified protocol for transdiagnostic treatment of emotional disorders compared with diagnosis-specific protocols for anxiety disorders: a randomized controlled trial. *JAMA Psychiatry* 74 875–884. 28768327 10.1001/jamapsychiatry.2017.2164 PMC5710228

[B3] Beard C. Björgvinsson T. (2014). Beyond generalized anxiety disorder: psychometric properties of the GAD-7 in a heterogeneous psychiatric sample. *J. Anxiety Disord.* 28 547–552. 10.1016/j.janxdis.2014.06.002 24983795

[B4] Becker K. D. Stirman S. W. (2011). The science of training in evidence-based treatments in the context of implementation programs: current status and prospects for the future. *Adm. Policy Ment. Health* 38 217–222. 21644028 10.1007/s10488-011-0361-0 PMC3565531

[B5] Chambless D. Hollon S. D. (1998). Defining empirically supported therapies. *J. Consult. Clin. Psychol.* 66 7–18. 10.1037/0022-006x.66.1.7 9489259

[B6] Chorpita B. Weisz J. R. Daleiden E. L. Schoenwald S. K. Palinkas L. A. Miranda J. (2013). Research network on youth mental health. Long-term outcomes for the child STEPs randomized effectiveness trial: a comparison of modular and standard treatment designs with usual care. *J. Consult. Clin. Psychol.* 81 999–1009. 10.1037/a0034200 23978169

[B7] Craigie M. A. Nathan P. (2009). A nonrandomized effectiveness comparison of broad-spectrum group CBT to individual CBT for depressed outpatients in a community mental health setting. *Behav. Ther.* 40 302–314. 10.1016/j.beth.2008.08.002 19647531

[B8] Deacon B. Abramowitz J. (2004). Cognitive and behavioral treatments for anxiety disorders: a review of meta-analytic findings. *J. Clin. Psychol.* 60 429–441. 10.1002/jclp.10255 15022272

[B9] DeRubeis R. J. Hollon S. D. Amsterdam J. D. Shelton R. C. Young P. R. Salomon R. M. (2005). Cognitive therapy vs medications in the treatment of moderate to severe depression. *Arch. Gen. Psychiatry* 62 409–416. 15809408 10.1001/archpsyc.62.4.409

[B10] Gloaguen V. Cottraux J. Cucherat M. Blackburn I. M. (1998). A meta-analysis of the effects of cognitive therapy in depressed patients. *J. Affect. Disord.* 49 59–72. 10.1016/s0165-0327(97)00199-7 9574861

[B11] Goldstein-Piekarski A. N. Williams L. M. Humphreys K. (2016). A trans-diagnostic review of anxiety disorder comorbidity and the impact of multiple exclusion criteria on studying clinical outcomes in anxiety disorders. *Trans. Psychiatry* 6:847. 10.1038/tp.2016.108 PMC4931606 27351601

[B12] Gyani A. Shafran R. Layard R. Clark D. M. (2013). Enhancing recovery rates: lessons from year one of IAPT. *Behav. Res. Ther.* 51 597–606. 10.1016/j.brat.2013.06.004 23872702 PMC3776229

[B13] Hansen N. B. Lambert M. J. Forman E. M. (2002). The psychotherapy dose-response effect and its implications for treatment delivery services. *Clin. Psychol.* 9 329–343. 10.1093/clipsy.9.3.329

[B14] Hofmann S. G. Asnaani A. Vonk I. J. Sawyer A. T. Fang A. (2012). The efficacy ofcognitive behavioral therapy: a review of meta-analyses. *Cogn. Ther. Res.* 36 427–440. 10.1007/s10608-012-9476-1 23459093 PMC3584580

[B15] Jacobson N. S. Truax P. (1991). Clinical significance: a statistical approach to defining meaningful change in psychotherapy research. *J. Consult. Clin. Psychol.* 59 12–19. 10.1037/0022-006x.59.1.12 2002127

[B16] Jonsson H. Thastum M. Arendt K. Juul-Sorensen M. (2015). Group cognitive behavioural treatment of youth anxiety in community based clinical practice: clinical significance and benchmarking against efficacy. *J. Anxiety Disord.* 35 9–18. 10.1016/j.janxdis.2015.06.009 26283461

[B17] Kazdin A. (2008). Evidence-based treatment and practice: new opportunities to bridge clinical research and practice, enhance the knowledge base, and improve patient care. *Am. Psychol.* 63 146–159. 10.1037/0003-066x.63.3.146 18377105

[B18] Kroenke K. Spitzer R. L. Williams J. B. (2001). The PHQ-9: validity of a briefdepression severity measure. *J. Gen. Int. Med*, 16 606–613. 10.1046/j.1525-1497.2001.016009606.x 11556941 PMC1495268

[B19] Kroenke K. Spitzer R. L. Williams J. B. Monahan P. O. Lowe B. (2007). Anxiety disorders in primary care: prevalence, impairment, comorbidity, and detection. *Ann. Int. Med.* 146 317–325. 17339617 10.7326/0003-4819-146-5-200703060-00004

[B20] Lakens D. (2013). Calculating and reporting effect sizes to facilitate cumulative science: a practical primer for t-tests and ANOVAs. *Front. Psychol.* 4:863. 10.3389/fpsyg.2013.00863 24324449 PMC3840331

[B21] Lilienfeld S. O. Ritschel L. A. Lynn S. J. Cautin R. L. Latzman R. D. (2013). Why many clinical psychologists are resistant to evidence-based practice: root causes and constructive remedies. *Clin. Psychol. Rev.* 33 883–900. 23647856 10.1016/j.cpr.2012.09.008

[B22] Loerinc A. G. Meuret A. E. Twohig M. P. Rosenfield D. Bluett E. J. Craske M. G. (2015). Response rates for CBT for anxiety disorders: Need for standardized criteria. *Clin. Psychol. Rev.* 42 72–82. 26319194 10.1016/j.cpr.2015.08.004

[B23] Nelson T. Steele R. (2007). Predictors of practitioner self-reported use of evidence-based practices: practitioner training, clinical setting, and attitudes toward research. *Adm. Policy Ment. Health* 34 319–330. 10.1007/s10488-006-0111-x 17268858

[B24] Plummer F. Manea L. Trepel D. McMillan D. (2016). Screening for anxiety disorders with the GAD-7 and GAD-2: a systematic review and diagnostic metaanalysis. *Gen. Hosp. Psychiatry* 39 24–31. 10.1016/j.genhosppsych.2015.11.005 26719105

[B25] Shadish W. R. Matt G. E. Navarro A. M. Phillips G. (2000). The effects of psychological therapies under clinically representative conditions: a meta-analysis. *Psychol. Bull.* 126 512–529. 10.1037/0033-2909.126.4.512 10900994

[B26] Shafran R. Clark D. Fairburn C. Arntz A. Barlow D. H. Ehlers A. (2009). Mind the gap: improving the dissemination of CBT. *Behav. Res. Ther.* 47 902–909. 10.1016/j.brat.2009.07.003 19664756

[B27] Shapiro D. A. Barkham M. Rees A. Hardy G. E. Reynolds S. Startup M. (1994). Effects of treatment duration and severity of depression on the effectiveness of cognitive-behavioral and psychodynamic-interpersonal psychotherapy. *J. Consult. Clin. Psychol.* 62 522–534. 8063978 10.1037/0022-006x.62.3.522

[B28] Spitzer R. L. Kroenke K. Williams J. B. Lowe B. (2006). A brief measure for assessing generalized anxiety disorder: the GAD-7. *Arch. Int. Med.* 166 1092–1097. 16717171 10.1001/archinte.166.10.1092

[B29] Stewart R. E. Chambless D. L. (2007). Does psychotherapy inform treatment decisions in private practice? *J. Clin. Psychol.* 63 267–281. 17211876 10.1002/jclp.20347

[B30] Stewart R. E. Chambless D. L. (2009). Cognitive-behavioral therapy for adult anxiety disorders in clinical practice: a meta-analysis of effectiveness studies. *J. Consult. Clin. Psychol.* 77 595–606. 10.1037/a0016032 19634954 PMC8022196

[B31] Tolin D. F. McKay D. Forman E. M. Klonsky E. D. Thombs B. D. (2015). Empirically supported treatment: recommendations for a new model. *Clin. Psychol.* 22 317–338. 10.1111/cpsp.12122

[B32] Waller G. (2009). Evidence-based treatment and therapist drift. *Behav. Res. Ther.* 47 119–127. 10.1016/j.brat.2008.10.018 19036354

[B33] Weisz J. R. Jensen-Doss A. Hawley K. M. (2006). Evidence-based youth psychotherapies versus usual clinical care: a meta-analysis of direct comparisons. *Am. Psychol.* 61 671–689. 10.1037/0003-066x.61.7.671 17032068

[B34] Westen D. Morrison K. (2001). A multidimensional meta-analysis of treatments fordepression, panic, and generalized anxiety disorder: an empirical examination of the status of empirically supported therapies. *J. Consult. Clin. Psychol.* 69 875–899. 10.1037/0022-006x.69.6.875 11777114

